# Full functional-length urethral sphincter- and neurovascular bundle preservation improves long-term continence rates after robotic-assisted radical prostatectomy

**DOI:** 10.1007/s11701-022-01408-7

**Published:** 2022-04-22

**Authors:** Benedikt Hoeh, Jan L. Hohenhorst, Mike Wenzel, Clara Humke, Felix Preisser, Clarissa Wittler, Marie Brand, Jens Köllermann, Thomas Steuber, Markus Graefen, Derya Tilki, Pierre I. Karakiewicz, Andreas Becker, Luis A. Kluth, Felix K. H. Chun, Philipp Mandel

**Affiliations:** 1 Department of Urology, University Hospital Frankfurt, Goethe University Frankfurt am Main, Theodor-Stern-Kai 7, 60590 Frankfurt am Main, Germany; 2 Cancer Prognostics and Health Outcomes Unit, Division of Urology, University of Montréal Health Center, Montréal, QC Canada; 3 Martini-Klinik Prostate Cancer Center, University Hospital Hamburg-Eppendorf, Hamburg, Germany; 4 Dr. Senckenberg Institute of Pathology, University Hospital Frankfurt, Frankfurt am Main, Germany; 5 Department of Urology, University Hospital Hamburg-Eppendorf, Hamburg, Germany; 6 Department of Urology, Koc University Hospital, Istanbul, Turkey

**Keywords:** Urinary continence, Urinary incontinence, Radical prostatectomy, FFLU, NVBP, Functional outcomes

## Abstract

The objective of the study was to test the impact of implementing standard full functional-length urethral sphincter (FFLU) and neurovascular bundle preservation (NVBP) with intraoperative frozen section technique (IFT) on long-term urinary continence in patients undergoing robotic-assisted radical prostatectomy (RARP). We relied on an institutional tertiary-care database to identify patients who underwent RARP between 01/2014 and 09/2019. Until 10/2017, FFLU was not performed and decision for NVBP was taken without IFT. From 11/2017, FFLU and IFT-guided NVBP was routinely performed in all patients undergoing RARP. Long-term continence (≥ 12 months) was defined as the usage of no or one safety- pad. Uni- and multivariable logistic regression models tested the correlation between surgical approach (standard vs FFLU + NVBP) and long-term continence. Covariates consisted of age, body mass index, prostate volume and extraprostatic extension of tumor. The study cohort consisted of 142 patients, with equally sized groups for standard vs FFLU + NVBP RARP (68 vs 74 patients). Routine FFLU + NVBP implementation resulted in a long-term continence rate of 91%, compared to 63% in standard RARP (*p* < 0.001). Following FFLU + NVBP RARP, 5% needed 1–2, 4% 3–5 pads/24 h and no patient (0%) suffered severe long-term incontinence (> 5 pads/24 h). No significant differences in patient or tumor characteristics were recorded between both groups. In multivariable logistic regression models, FFLU + NVBP was a robust predictor for continence (Odds ratio [OR]: 7.62; 95% CI 2.51–27.36; *p* < 0.001). Implementation of FFLU and NVBP in patients undergoing RARP results in improved long-term continence rates of 91%.

## Introduction

Radical prostatectomy (RP) is one of the main definite treatment modalities for clinically localized and locally advanced prostate cancer (PCa) and provides favorable cancer control [1–5]. Despite the fact that cancer control represents the unnegotiable central aim in RP, ensuring acceptable functional outcomes is of utmost importance, too [2, 6–8]. Among those, postprostatectomy urinary incontinence is a frequent complication occurring in 4–20% of patients undergoing RP, depending on the various definitions of continence and follow-up time [1, 2, 9, 10]. Previous studies have demonstrated that urinary incontinence impairs drastically health-related quality of life of affected patients, depicted by a strikingly increased risk of developing mental health issues [8, 11, 12]. Most commonly, sphincter injury and bladder damage are anatomically associated with postoperative urinary incontinence [13–15]. Schlomm et al. introduced a surgical technique for full functional-length urethra preservation (FFLU) during RP and reported significantly better short-term (1 week after catheter removal) continence rates (*p* < 0.001) relatively to patients not undergoing FFLU at RP [10]. On the basis of these findings, a new standard of care, consisting of FFLU and neurovascular bundle preservation (NVBP) with intraoperative frozen section technique (IFT), was introduced in 11/2017 at our institution [16, 17]. Previously, Theissen et al. demonstrated that the introduction of this new standard of care resulted in promising very early continence rates after catheter removal [17]. Whether the implementation of the new standard of care would be still apparent in long-term continence rates, is however uncertain. We addressed this void in the current study. We hypothesized that implementation of FFLU and NVBP as the new standard of care would result in improved long-term continence rates in RARP patients. We compared long-term continence rates of robotic-assisted radical prostatectomy (RARP) patients before and after implementation of the new standard of care, consisting of FFLU and IFT-NVBP (FFLU + NVBP) at our institution.

## Materials and methods

### Study population

From 01/2014 to 09/2019, 296 patients treated with RARP were retrospectively identified from our prospective institutional database. Of those, long-term continence data (≥ 12 months) were available for 142 patients (48.0%). Indication for RARP was histologically confirmed prostate cancer. Exclusion criteria consisted of neoadjuvant (androgen deprivation) therapy (*n* = 3) and clinical suspicion of metastases (*n* = 3).

Before 11/2017, RARP was performed without FFLU and decision for NVBP was taken without routine IFT at the Department of Urology, University Hospital Frankfurt. The indication for NVBP was assessed using preoperative data from prostate magnetic resonance imaging and D’Amico risk classification [18], and according to the nomograms of Kattan et al. [19] and Steuber et al. [20]. In 11/2017, FFLU in combination with IFT-NVBP was introduced at the Department of Urology as a new standard of care, as previously described. If IFT demonstrated positive surgical margins at the site of neurovascular bundle resection, secondary resection of the affected site was routinely performed [10, 16, 20, 21].

Stratification according to the surgical approach before 11/2017, namely standard RARP and after 11/2017, namely FFLU + NVBP, resulted in two equally sized groups of 68 vs 74 patients, respectively (Table 1).

**Table 1:** Descriptive patient and tumor characteristics of 142 patients who underwent robotic-assisted radical prostatectomy and available long-term continence information between 01/2014 and 09/2019, stratified according to standard vs routine implementation of full functional-length urethral sphincter preservation (FFLU) and neurovascular bundle preservation (NVBP)

	Overall, *n* = 142	Standard*, *n* = 68	FFLU + NVBP, *n* = 74	*P* value
Age in years, Median (IQR)	66 (61, 71)	66 (62, 71)	66 (59, 71)	0.7
PSA in mg/ml, Median (IQR)	6.8 (5.4, 10.0)	6.8 (5.4, 11.6)	7.3 (5.5, 9.6)	0.6
Body mass index in kg/m^2^, Median (IQR)	25.9 (24.2, 28.5)	26.1 (24.0, 28.6)	25.9 (24.5, 28.4)	0.9
Body mass index grouped in kg/m^2^, *n* (%)				
≤ 25	50 (36%)	24 (36%)	26 (36%)	0.8
25–30	63 (45%)	31 (47%)	32 (44%)	
≥ 30	26 (19%)	11 (17%)	15 (21%)	
Intraoperative blood loss in ml, Median (IQR)	300 (200, 400)	200 (200, 300)	300 (200, 400)	0.2
Operation time in min, Median (IQR)	238 (189, 286)	229 (177, 295)	241 (200, 285)	0.3
Prostate volume in cm^3^, Median (IQR)	36 (30, 49)	34 (27, 45)	40 (30, 50)	0.2
Gleason grade group Biopsy-specimen, *n* (%)				0.3
I	35 (25%)	20 (29%)	15 (20%)	
II	64 (45%)	29 (43%)	35 (47%)	
III	24 (17%)	9 (13%)	15 (20%)	
IV	13 (9.2%)	5 (7.4%)	8 (11%)	
V	6 (4.2%)	5 (7.4%)	1 (1.4%)	
D’Amico risk classification, *n* (%)				0.093
Low	24 (17%)	13 (20%)	11 (15%)	
Intermediate	88 (63%)	37 (56%)	51 (69%)	
High	28 (20%)	16 (24%)	12 (16%)	
Gleason grade group RP-specimen, *n* (%)				0.3
I	29 (20%)	13 (19%)	16 (22%)	
II	72 (51%)	33 (49%)	39 (53%)	
III	21 (15%)	8 (12%)	13 (18%)	
IV	7 (4.9%)	5 (7.4%)	2 (2.7%)	
V	13 (9.2%)	9 (13%)	4 (5.4%)	
Nerve sparing, *n* (%)				< 0.001
Bilateral	99 (73%)	35 (57%)	64 (86%)	
Unilateral	12 (8.9%)	6 (9.8%)	6 (8.1%)	
None	24 (18%)	20 (33%)	4 (5.4%)	
Positive surgical margin, *n* (%)				0.4
R0	104 (73%)	52 (76%)	52 (70%)	
R1	36 (25%)	16 (24%)	20 (27%)	
Rx	2 (1.4%)	0 (0%)	2 (2.7%)	
pT-stage, *n* (%)				0.6
pT2a	9 (6.4%)	5 (7.5%)	4 (5.4%)	
pT2b	4 (2.8%)	2 (3.0%)	2 (2.7%)	
pT2c	78 (55%)	35 (52%)	43 (58%)	
pT3a	35 (25%)	15 (22%)	20 (27%)	
pT3b	13 (9.2%)	9 (13%)	4 (5.4%)	
pT4	2 (1.4%)	1 (1.5%)	1 (1.4%)	
Extraprostatic extension of tumor, *n* (%)				0.7
T2	91 (65%)	42 (63%)	49 (66%)	
T3/T4	50 (35%)	25 (37%)	25 (34%)	
pN-stage, *n* (%)				0.037
pN0	127 (89%)	59 (87%)	68 (92%)	
pN1	8 (5.6%)	7 (10%)	1 (1.4%)	
pNx	7 (4.9%)	2 (2.9%)	5 (6.8%)	

All values are median (IQR) or frequencies (%)

*FFLU* full functional-length urethral sphincter preservation, *NVBP* neurovascular bundle preservation, *IQR* interquartile range, *PSA* prostate-specific antigen

*NVBP was performed when oncological reasonable, but without intraoperative frozen section technique (IFT)

All surgeons, who performed RARP in the current study period, were experienced surgeons trained in high-volume prostate cancer centers. Written informed consent was obtained from all patients, and the study was approved by the institutional review boards of the University Cancer Centre Frankfurt and the Ethical Committee at the University Hospital Frankfurt.

### Outcome measurements

Long-term continence status was ascertained based on voluntary self-reported standardized questionnaires, as previously described [2, 22]. Long-term continence was defined as no or one safety-pad usage within 24 h at least 12 months after RARP, whereas a higher number of pads was considered incontinent. Usage of pads was grouped as followed: 0–1 safety, 1–2, 3–5 and > 5 pads within 24 h. If two follow-up assessments were available (*n* = 1), the more mature assessment was considered for further analyses. Stratification was performed according to standard vs FFLU + NVBP RARP, respectively. Subsequently, additional subgroup analyses were conducted, which specifically relied on surgeons who performed both standard and subsequently FFLU + NVBP approach over time.

### Statistical analyses

Descriptive statistics included frequencies and proportions for categorical variables. Medians and interquartile ranges (IQR) were reported for continuously coded variables. The chi-squared test examined the statistical significance of the differences in proportions while the Kruskal–Wallis test was used to examine differences in medians.

Uni- and multivariable logistic regression models tested the relationship between surgical approach (standard vs FFLU + NVBP) and long-term urinary continence, defined as no or one safety pad usage within 24 h. Covariates consisted of age at RARP (≤ 60 vs 61–69 vs ≥ 70 years), body mass index (BMI) (< 25 vs 25–30 vs > 30 kg/m^2^), prostate volume (≤ 40 vs > 40 ml), nerve-sparing (no vs uni/bilateral), and extraprostatic extension of tumor (pT2 vs pT3/4). For all statistical analyses R software environment for statistical computing and graphics (version 3.4.3) was used. All tests were two-sided with a level of significance set at *p* < 0.05.

## Results

### Descriptive characteristics of the study population

In total, 142 patients represented the focus of the current analyses (Table 1). Of those, 74 patients (52%) underwent FFLU + NVBP, whereas 68 patients (48%) standard RARP, respectively. In the overall cohort, median age was 66 years (IQR: 61–71), median PSA 6.8 ng/ml (IQR: 5.4–10.0) and median BMI 25.9 kg/m^2^ (IQR: 24.2–28.5) and did not differ between both groups. Median operation time was 241 vs 229 min for FFLU + NVBP vs standard RARP (*p* = 0.3). Nerve sparing (uni/bilateral) was performed in 94% vs 67% patient undergoing FFLU + NVBP vs standard RARP, respectively (*p* < 0.001). Final histopathological examination reported in 35% of all patients extraprostatic extension of the tumor and did not differ between both groups (*p* = 0.7).

### Long-term continence rates

Long-term continence rates were 91% vs 63% in FFLU + NVBP vs standard RARP, respectively (*p* < 0.001), applying definition of continence as no or one safety pad use within 24 h at least 12 months after RARP (Table 2). Rates for usage of 1–2, 3–5, and > 5 pads within 24 h were: 5.4 vs 17.6%, 4.1 vs 11.8%, and 0 vs 7.4% for FFLU + NVB vs standard RARP (p < 0.001) with a median follow-up time of 450 days (IQR: 400–582) vs 1308 days (IQR: 856–1545) for FFLU + NVB vs standard RARP patients (*p* < 0.001). Results remained qualitatively and quantitatively unchanged in subgroup analyses solely focusing on surgeons who performed RARP prior and after implementation of standard FFLU + NVBP in 11/2017.

**Table 2:** Long-term continence rates of 142 patients treated with robotic-assisted radical prostatectomy between 01/2014 and 09/2019, stratified according to implementation of full functional-length urethral sphincter preservation (FFLU) and neurovascular bundle preservation (NVBP)

	Overall, *n* = 142	Standard*, *n* = 68	FFLU + NVBP, *n* = 74	*P* value
Long-term continence, *n* (%)				
Yes	110 (77.5%)	43 (63.2%)	67 (90.5%)	< 0.001
No	32 (22.5%)	25 (36.8%)	7 (9.5%)	
Numbers of pads/24 h, *n* (%)				
0–1(safety pad)	110 (77.5%)	43 (63.2%)	67 (90.5%)	< 0.001
1–2	16 (11.3%)	12 (17.6%)	4 (5.4%)	
3–5	11 (7.7%)	8 (11.8%)	3 (4.1%)	
> 5	5 (3.5%)	5 (7.4%)	0 (0%)	

All values are median (IQR) or frequencies (%)

*NVBP was performed when oncological reasonable, but without intraoperative frozen section technique

### Uni- and multivariable logistic regression models

In univariable logistic regression models, FFLU + NVBP was a statistically significant predictor for long-term urinary continence, yielding an odds ratio (OR) of 5.56 [95% CI 2.32–14.97; *p* < 0.001] (Table 2). Besides age ≥ 70 years, which was associated with less chances of urinary continence [OR: 0.17; 95% CI 0.04–0.57; *p* = 0.009], solely nerve sparing was associated with a significant higher chance of urinary continence [OR: 3.25; 95% CI 1.24–8.37; *p* = 0.02]. Neither BMIor extraprostatic extension nor prostate volume were significant predictors for urinary continence in univariable analyses. After adjustment in multivariable logistic regressions models, FFLU + NVBP remained a strong predictor for long-term urinary continence [OR: 7.62; 95% CI 2.51–27.36; *p* < 0.001]. Furthermore, age ≥ 70 [OR: 0.12; 95% CI: 0.02–0.48; *p* = 0.006] remained to be associated with less chances of urinary continence in multivariable analyses. All other covariates failed to reach statistically significant predictor status (Table 3).

**Table 3:** Uni- and multivariable logistic regression models predicting long-term (≥ 12 months) urinary continence in 142 patients treated with robotic-assisted radical prostatectomy

	Univariable				Multivariable			
	Odds ratio	95% CI		*P* value	Odds ratio	95% CI		*P* value
		2.5%	97.5%			2.5%	97.5%	
Surgical approach								
Standard*	*Ref*				*Ref*			
FFLU + NVBP	5.56	2.32	14.97	< 0.001	7.62	2.51	27.36	< 0.001
Body mass index in kg/m^2^								
< 25	*Ref*				*Ref*			
25–30	0.44	0.17	1.09	0.083	0.33	0.10	1.00	0.06
≥ 30	0.80	0.24	2.92	0.72	0.45	0.09	2.20	0.31
Age in years								
≤ 60	*Ref*				*Ref*			
61–69	0.35	0.08	1.20	0.13	0.50	0.10	2.00	0.35
≥ 70	0.17	0.04	0.57	0.009	0.12	0.02	0.48	0.006
Nerve sparing								
None	*Ref*				*Ref*			
Uni/bilateral	3.25	1.24	8.37	0.02	1.29	0.37	4.33	0.68
Extraprostatic extension								
No	*Ref*				*Ref*			
Yes	0.99	0.44	2.36	1.00	1.17	0.41	3.49	0.77
Prostate volume in ml								
≤ 40 ml	*Ref*				*Ref*			
> 40 ml	1.18	0.52	2.76	0.70	0.71	0.23	2.12	0.53

Urinary continence was defined by usage of no or one safety pad within 24 h. Extraprostatic extension of the tumor was defined by pT3/pT4 in final RP-specimen

*RARP* robotic-assisted radical prostatectomy, *FFLU* full functional-length urethral sphincter preservation, *NVBP* neurovascular bundle preservation, 95% *CI* 95% confidence interval

*NVBP was performed when oncological reasonable, but without intraoperative frozen section technique

## Discussion

We hypothesized that implementation of FFLU and NVBP as the new standard of care would result in improved long-term continence rates in RARP patients. Previous anatomical and functional studies have demonstrated that a substantial functional part of the urethral sphincter is located intraprostatically between the colliculus seminalis and apex [10, 23, 24]. Bearing in mind that the apex shape varies widely among patients, up to 40% of the functional part of the urethral sphincter is covered by parenchymal apex tissue [25–27]. As a consequence, meticulous preservation of the full functional-length of the urethra would include preserving a substantial part of the urethral sphincter complex as well [10]. Furthermore, several studies have reported improved continence rates in patients undergoing RARP with preservation of the neurovascular bundles [28]. Conversely, Michl et al. demonstrated that the observed beneficial effect in continence was more likely attributed to the meticulous apical dissection during nerve-sparing technique rather than the preservation of the neurovascular bundle preservation itself [29]. To address this void, we tested the potential beneficial effect of FFLU and/or NVBP on long-term continence rates and compared continence rates of RARP patients before and after implementation of the new standard of care, consisting of FFLU + NVBP at our institution and made some noteworthy findings.

First and foremost, long-term continence rates were 91% following the implementation of the new standard of care compared to 63% prior to the change in care policy in RARP. These findings indicate that the implementation of FFLU + NVBP had a substantial effect not only on the short-term continence (previously reported by Theissen et al.), but also translate into an improvement in long-term continence [17]. Different aspects have to be taken into account while interpreting these results. Even though that the vast majority of patients´ and tumor characteristics (Age, BMI, blood-loss, prostate volume, D’Amico risk classification) did not differ between both study cohorts, some variables differed significantly and should be interpreted accordingly. As stated above, nerve sparing approach was based on preoperative assessments in patients undergoing RARP before 11/2017, resulting in 67% patients receiving either uni or bilateral nerve sparing. By contrast after 11/2017, nerve sparing was performed as a standard of care in combination with routinely usage of intraoperative frozen, yielding a higher percentage of 95% receiving either uni or bilateral nerve sparing. Therefore, to further analyze whether FFLU or NVB account for the higher chance of long-term continence after 11/2017, multivariable logistic regression models were additionally adjusted for nerve sparing performance. Hereby, implementation of FFLU remained quantitatively and qualitatively virtually unchanged as the strongest predictor for long-term continence [data not shown; multi. OR: 7.62; 95% CI 2.51–27.36; *p* < 0.001]. Interestingly and in line with findings by Michl et al., (uni/bilateral) NVBP failed to reach independent predictor status in multivariable logistic regression [29]. These findings underline the assumption that the beneficial effect most likely originates from the meticulous dissection while nerve sparing is performed rather than the nerve bundle preservation itself [29].

Second, besides very important differences in long-term continence rates, severity of incontinence and its distribution differed significantly between patients treated with standard vs FFLU + NVBP RARP, respectively. It is of note that less than 5% of patients being treated with FFLU + NVBP reported a usage of three or more pads within 24 h (Table 2). Conversely, the rates for three or more pads in patients with standard RARP were significantly higher (19%, *p* < 0.001). Irrespectively by the limited numbers of events within the subgroup of incontinent patients, the current data suggest that FFLU + NVBP will have positive effects not only on the rates of continence, but also positively influences the level of severity substantially.

Third, implementation of new standard of care translated in a very timely improvement in long-term continence rates after its initial implementation as new standard of care. Taking into consideration that the current data for long-term continence rates rely on solely patients being treated within the first 21 months after FFLU + NVBP implementation, the findings demonstrate that results of implementation of FFLU + NVBP will be depictable in a very timely manner.

The current study is not devoid of limitations. First and foremost are the limitations inherent to the retrospective nature of the study and the limited sample size. Second, a potential bias regarding the extent of postsurgical pelvic-floor training cannot be ruled out. However, all patients were strongly encouraged to seek professional pelvic-floor training for urinary continence recovery and were already instructed during their in-patient stay. Third, current findings rely on patients solely treated with RARP. Whether these findings can be unrestrained transferred to open RP, cannot be drawn from the current study. Moreover, whether the current findings are transferable to different surgical approaches and techniques (for example Retzius-sparing approach)  could not be addressed within the current manuscript [30, 31]. Fourth, in cancer-related surgery, such as RALP in the current study, oncological outcomes may not be neglected to improve functional outcomes. We acknowledge that in the current manuscript, positive surgical margins did not differ between standard vs FFLU/NVBP approach. The less beneficial effect of FFLU/NVBP approach on positive surgical margins is most likely explainable due to sample size limitations in the current manuscript. It is of interest that previous studies, relying on a larger study cohort, demonstrated that implementation of IFT resulted in a statistically significant lower rate of positive surgical margins [16, 32]. Furthermore, Schlomm et al., relying on 5392 RP patients treated with an IFT-approach (‘NEUROSAFE’), reported that IFT did not have a negative impact on biochemical-recurrence free survival, additional to lower rates of positive surgical margins [32]. Fifth, RARP were performed by several surgeons over the study period, differences in experience level among the surgeons might have been present. To test for such potential bias, we explicitly relied on data of surgeons who performed RARP before and after the implementation of FFLU + NVPB at our institution. Hereby, continence rates following FFLU + NVBP RARP (96%) remained substantial higher compared to standard RARP (63%). Therefore, changes in continence rates are unlikely be solely driven by differences in surgeons experience levels. Finally, all limitations that are inherently linked to data derived from voluntary, self-questionnaire reporting account for the current study.

## Conclusion

Implementation of FFLU and NVBP in patients undergoing RARP results in improved long-term continence rates of 91%. Additionally, less severe incontinence was recorded for patients undergoing FFLU and NVBP compared to standard RARP.

## Data Availability

All datasets generated for this study are included in the manuscript (supplementary files). B.H. had full access to all the data in the study and takes responsibility for the integrity of the data and the accuracy of the data analysis.
